# The Effect of Different Surface Treatments on the Roughness, Translucency, and Staining of 3D‐Printed Occlusal Device Materials

**DOI:** 10.1111/jerd.13476

**Published:** 2025-04-03

**Authors:** Silvia Rojas Rueda, Hanna Sepsick, Mohammed Hammamy, Amir H. Nejat, Edwin Kee, Nathaniel C. Lawson

**Affiliations:** ^1^ Resident, Department of Clinical and Community Sciences The University of Alabama at Birmingham School of Dentistry Birmingham Alabama USA; ^2^ Dental Student The University of Alabama at Birmingham School of Dentistry Birmingham Alabama USA; ^3^ Assistant Professor, Division of Prosthodontics Louisiana State University School of Dentistry New Orleans Louisiana USA; ^4^ Associate Professor, Department of Clinical and Community Sciences The University of Alabama at Birmingham School of Dentistry Birmingham Alabama USA

**Keywords:** 3D printing, NightGuard, splints, surface roughness, translucency

## Abstract

**Objectives:**

To compare surface treatments (as‐printed, optical polish, resin‐coated, polished) of two 3D‐printed occlusal device materials (KeySplint Soft and NightGuard Flex 2) cured with or without glycerin for surface roughness, translucency, and coffee staining.

**Materials and Methods:**

Discs (2 mm thick) from two 3D‐printed occlusal resins (KeySplint Soft and NightGuard Flex 2) were printed using a DLP 3D printer (SprintRay Pro 95), cleaned (ProWash S), and cured (ProCure 2) with or without glycerin. Some specimens were printed in an optical polish tank. Specimens were either as‐printed, resin‐coated, or polished. Reference milled (ProArt CAD Splint) and heat‐cured (Excel Formula Heat Cure Denture Base Material) specimens were also prepared. Surface roughness was analyzed using a contact profilometer. Translucency was measured using a spectrophotometer. Staining was evaluated after 24 days in coffee at 37°C. Data were analyzed with ANOVA and Tukey post hoc tests.

**Results:**

Surface treatments and glycerin curing showed significant differences (*p* < 0.01). Polishing and resin‐coating produced the smoothest surfaces. Optical polish tanks improved smoothness. Polishing increased translucency. Glycerin curing reduced staining except in polished specimens. Milled materials stained less than 3D‐printed materials.

**Conclusions:**

Polishing and resin‐coating optimized roughness and translucency. Polishing or curing glycerin optimized stain resistance.

**Clinical Significance:**

3D‐printed occlusal devices should have their external surfaces polished or resin‐coated and receive a final cure in glycerin to prevent staining of their internal as‐printed surface. Printing in a tank with an optical polish can help to improve the roughness and translucency of the internal surface of an occlusal device.

## 1 Introduction

As occlusal devices may need to be worn during the day for TMD treatments or awake bruxism [1, 2, 3, 4], occlusal device materials should have esthetic qualities, including translucency and stain resistance. Occlusal devices may be fabricated from conventional methods (lost wax, thermoformed, sprinkle‐on), milling, or three‐dimensional (3D) printing [5]. The 3D‐printing fabrication technique may result in microscopic voids between layers and a lower degree of resin conversion, both of which may contribute to increased water sorption [6]. Additionally, immediately after fabrication, 3D‐printed occlusal device materials exhibit greater roughness than milled or conventionally fabricated materials, particularly when printing at 90° to the build plate [7]. As a result, water‐based staining could be more common in 3D‐printed materials. A review indicated that 3D‐printed materials generally exhibited more staining than milled materials, although this finding was limited to 3D‐printed crown materials [8]. A laboratory study found that a 3D‐printed occlusal device material experienced more staining than a conventionally fabricated material [9]. No previous studies have compared stain resistance or translucency of 3D printed to conventional and milled occlusal device materials.

The translucency and stain resistance of a material are influenced by its surface properties. Although the intaglio surface of an occlusal device is typically left unadjusted to maintain proper fit, the external surface can be polished or resin‐coated. Aside from improving patient comfort and plaque accumulation, polishing can enhance both stain resistance and translucency [10, 11, 12, 13]. Increased roughness reduces translucency by altering the direction and incidence of light on the material's surface [12]. Additionally, polishing may minimize stain accumulation. A previous study found that polished 3D‐printed materials showed less staining compared to unaltered materials, likely due to the removal of surface features created during printing [13]. The roughness of an occlusal device is influenced by the polishing process used. For instance, previous studies found that a 3D‐printed material had an as‐printed roughness average (Ra) ranging from 0.335 to 2.1 μm, depending on the print orientation. This roughness could be reduced to between 0.06 and 0.87 μm using laboratory polishing brushes and down to 0.06 μm with a polishing wheel [7, 14]. Another technique for smoothing 3D‐printed materials is resin coating, where the same resin used in the printing process is applied to the surface and then re‐cured. A previous study indicated that although resin coating can reduce the roughness of a printed material, it is not as effective as polishing [15].

Additionally, occlusal device materials can be immersed in glycerin during the curing process to enhance their degree of conversion. Glycerin serves as an oxygen barrier, preventing the inhibition of resin polymerization by minimizing oxygen diffusion to the resin surface [16]. Curing in glycerin can improve the stain resistance of 3D‐printed materials [17].

One innovation in 3D printing is the use of a printing tank equipped with an optical polish layer that facilitates a smoother final surface on printed objects. Although manufacturers do not provide specific details about the technology used in this layer, it likely includes an intensity‐controlling film designed to minimize staircase artifacts associated with voxels in digital light processing (DLP) printing. When curing resin in a tank of a 3D printer, the solidification of each voxel is governed by the intensity of light it receives. If the light intensity is lowered, the microscopic surface texture of a printed object may have incomplete solidification of the voxel, reducing staircase artifacts [18].

The purpose of this study was to compare the roughness, translucency, and stain resistance of 3D‐printed occlusal device materials with different surface treatments. The first null hypothesis was that there would be no difference in the roughness, translucency, and stain resistance of the different 3D‐printed occlusal device materials after various surface treatments (as‐printed, optical polish, resin‐coated, and polished) or curing in glycerin. No previous studies have compared different surface treatments or curing in glycerin for improving the esthetic properties specifically for occlusal device materials. The second null hypothesis was that there would be no difference in the roughness, translucency, and stain resistance of 3D‐printed, milled, and conventional occlusal device materials.

## 2 Materials and Methods

### 2.1 Specimen Fabrication

A sample size of 10 was chosen for each group due to the low expected variability in measurements and the large differences expected between groups. Disc‐shaped specimens (2 mm thickness × 15 mm in diameter) were fabricated from two types of 3D‐printed occlusal device materials: KeySplint Soft (Keystone Dental Inc.; Burlington, MA, USA) and NightGuard Flex 2 (SprintRay; Los Angeles, CA, USA). The print design for the specimens was generated in RayWare Version 2.9.1 (SprintRay) with the circular face oriented 90° to the build plate to avoid supports on the critical areas of the specimens. The placement of supports on the flat surface of the specimens would have required grinding and eliminated the texture present on the surfaces of the as‐printed specimens. All specimens were printed on a digital light projection (DLP) printer (Pro 95, SprintRay). Resin was mixed and poured into a standard resin tank (Pro95 resin tank, SprintRay) for most specimens except those printed in a resin tank featuring an optical polish (Pro 95 resin tank with optical polish, SprintRay; optical polish group). Specimens were printed with supports at a layer thickness of 100 μm. Post‐processing was performed by washing in 99% isopropyl alcohol in a 2‐step washing device (ProWash, SprintRay) and curing in a light cure unit (ProCure 2, SprintRay) as per manufacturer recommendations. Specimens in the as‐printed group received no further treatment. In order to avoid inter‐operator variability, all surface finishing was performed by one operator.

Specimens in the resin‐coated group were coated with 1 even layer of their respective resin using a brush (Benda Brush, Centrix; Shelton, CT, USA) prior to placement in the cure unit. The brush was dipped one time into a small bowl of resin and used to paint a thin layer onto the surface of one side of the specimen. The specimen was cured, and then, one coat was applied on the opposite side of the specimen. The specimens were then cured again. Both sides of the specimens were coated to eliminate the effects of the as‐printed side in translucency and staining testing.

Specimens in the polished group were polished using the procedure and products from a commercially available kit for polishing 3D‐printed occlusal devices (KeyPolish Kit, KeyPrint, Keystone Industries; Gibbstown, NJ, USA). Specimens were first ground to 320‐grit SiC paper as that provided a roughness similar to the medium Scotch Brite polisher present in the kit while leaving the surface flat [7]. Specimens were then wet polished with coarse then fine pumice (Lab Pumice, Coarse and Fine, Henry Schein; Melville, NY, USA) on a laboratory cloth polishing wheel (4 × 42 muslin buff, KeyPrint). Specimens were further polished with polishing compound (Tripoli Polishing Compound and Beige Paste Polish Bar, KeyPrint) on a new cloth polishing wheel. Both sides of the specimens were polished to eliminate the effects of the as‐printed side in translucency and staining testing.

**FIGURE 1:**
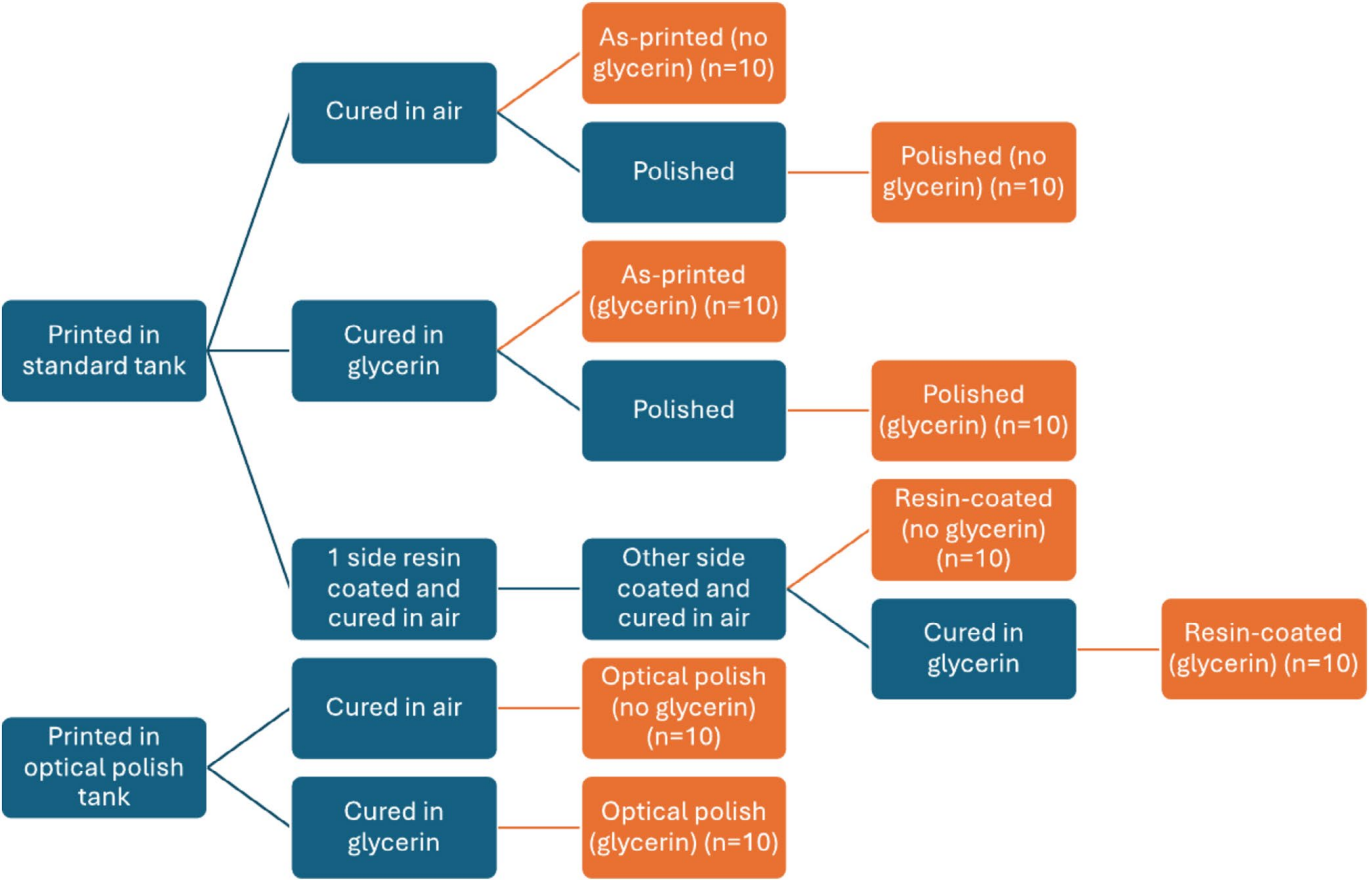
Study design.

In order to test the effects of curing in glycerin, additional specimens from each group (as‐printed, optical polish, resin‐coated, polished) were placed in a transparent bowl of glycerin (99.7% glycerin, Chemworld; Atlanta, GA, USA) and cured in the light cure unit. As‐printed and optical polished specimens were cured only once in glycerin. Resin‐coated specimens were cured in glycerin following the final cure of the second layer of resin coating. Polished specimens were cured in glycerin prior to polishing. A diagram of the study design is presented in Figure 1.

**FIGURE 2:**
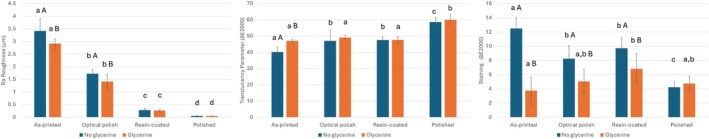
KeySplint Soft roughness, translucency, and staining. Different lowercase letters represent significant differences between different surface treatments (comparisons only within glycerin curing group). Different uppercase letters represent significant differences between different glycerin curing protocols (comparisons only within surface treatment group).

To test the second hypothesis, additional specimens of each 3D‐printed material were produced. Milled specimens were produced from ProART CAD Splint (Ivoclar; Schaan, Lichtenstein) using a 5‐axis milling unit (inLab MC X5, Dentsply Sirona; Charlotte, NC, USA), and heat‐polymerized specimens were created using Excel Formula Heat Cure Denture Base Material (St. George Technology; St. George, UT, USA) through traditional flasking, boiling‐out, and polymerization procedures in boiling water. All specimens were wet polished to 600‐grit silicon carbide abrasive paper to standardize their surface (Table 1).

**TABLE 1:** Materials evaluated in this study.

Type of material	Brand name	Manufacturer	Composition^a^
3D printed (flexible)	KeySplint Soft	KeyStone	Proprietary resins
3D printed (flexible)	NightGuard Flex 2	SprintRay Inc.	Methacrylate oligomer, acrylate monomer, methacrylate monomer, cyclohexanol 3,3,5‐trimethyl, phosphine oxide
Milled	ProART CAD Splint	Ivoclar AG	Polymethyl methacrylate
Heat‐polymerized	Excel Fromula Heat Cure Denture Base Material	St. George Technology	Methyl methacrylate, ethylene glycol dimethacrylate

^a^ Based on manufacturer's safety data sheet.

### 2.2 Surface Roughness

Surface roughness (Ra) measurements were conducted on all specimens by one operator using a contact profilometer (Surftest SJ‐210, Mitutoyo; Kawasaki, Japan) calibrated according to the manufacturer's guidelines. To minimize variability, two distinct points on each specimen were measured and averaged following ISO 4287 standards for profilometer settings. An initial scan was performed on the specimens in order to determine an approximate roughness value. Based on the initial scan, a sample length of 4 mm (Ra > 0.01) or 1.25 mm (Ra < 0.01) and a cut‐off filter of 0.8 mm (Ra > 0.01) or 0.25 mm (Ra < 0.01) were utilized. Where print lines could be observed (as‐printed and optical polish groups), the surface roughness was measured perpendicular to the print lines.

### 2.3 Translucency and Stain Resistance

L*a*b* values were measured with a spectrophotometer (CM‐700d, Konica Minolta; Ramsey, NJ) by one operator, with the specimens positioned against white and black calibrated tiles. The specimens were stored dry for 1 week in ambient light prior to the initial color measurements to allow any photobleaching of residual initiator to occur prior to measurement. The color change was quantified using Δ*E*2000, derived from the color difference between the white and black backgrounds, which represents the translucency parameter.

The same specimens were subsequently immersed in coffee for 28 days at 37°C. The coffee solution was prepared by dissolving 2 g of Folgers Classic Roast Instant Coffee (J.M. Smucker; Orrville, OH) in 6 fl. oz. of hot water. Specimens were laid flat in bags of coffee that were not refreshed or stirred. L*a*b* measurements were then taken in the same orientations as the initial readings, using the white calibrated tile as a reference. The color change was calculated using Δ*E*2000, based on the difference between the original and stained L*a*b* values.

### 2.4 Scanning Electron Microscopy

Representative specimens were analyzed with a scanning electron microscope (SEM, Quanta FEG 650, FEI; Hillsboro, OR). The specimens were secured onto SEM stubs with conductive tape and then gold‐coated using a vacuum sputter coater. Examination was performed using the secondary electron imaging mode.

### 2.5 Statistical Analysis

Data for surface roughness, translucency, and stain resistance were analyzed to assess statistically significant differences among groups. Individual two‐way ANOVAs for each 3D‐printed material were performed for each property to identify the effects of surface treatment and glycerin curing using SPSS software version 24 (IBM; Armonk, NY). A one‐way ANOVA of the polished occlusal device materials was performed for each property to identify differences in the materials. When significant differences were detected, Tukey post hoc tests were applied to further evaluate pairwise comparisons between specific groups. All analyses were performed with a significance level set at 5% (*p* < 0.05). A post hoc power analysis was conducted to determine the power for each ANOVA test at a significance level of 0.5 (*β* = 0.2) and effect size of 0.4 using G*Power (Heinrich–Heine‐Universität; Düsseldorf, Germany).

## 3 Results

**FIGURE 3:**
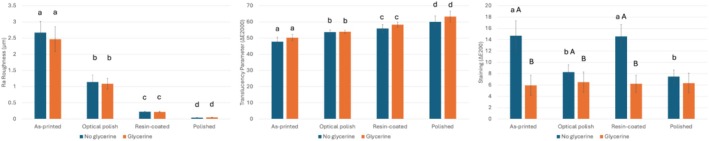
NightGuard Flex roughness, translucency, and staining. Different lowercase letters represent significant differences between different surface treatments (comparisons only within glycerin curing group). Different uppercase letters represent significant differences between different glycerin curing protocols (comparisons only within surface treatment group).

**FIGURE 4:**
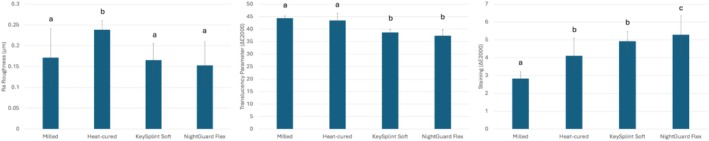
Different occlusal device material roughness, translucency, and staining. Different lowercase letters represent significant differences between different materials.

The post hoc power analysis determined that the study was powered at 70%. For KeySplint Soft, the two‐way ANOVA determined that surface treatment, glycerin curing, and their interaction were all significant (*p* < 0.01) for each property. For NightGuard Flex, the two‐way ANOVA determined that the surface treatment was significant for each property (*p* < 0.01), curing in glycerin and the interaction between surface treatment and curing in glycerin was only significant for staining (*p* < 0.01). For the comparison of different occlusal device materials, the one‐way ANOVA determined significant differences between materials for all properties (*p* < 0.01). The means, standard deviations, and statistical differences between groups for each property are presented in Figures 1, 2, 3, 4.

**FIGURE 5:**
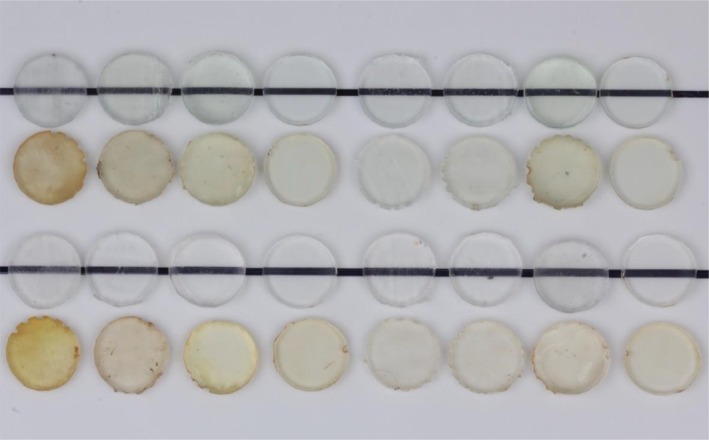
Photograph of representative specimens of (top to bottom): KeySplint Soft unstained, KeySplint Soft stained, NightGuard Flex unstained, and NightGuard Flex stained after different surface treatments and curing protocols (left to right): As‐printed, optical polish, resin‐coated, polished, as‐printed with glycerin cure, optical polish with glycerin cure, resin‐coated with glycerin cure, and polished with glycerin cure.

**FIGURE 6:**
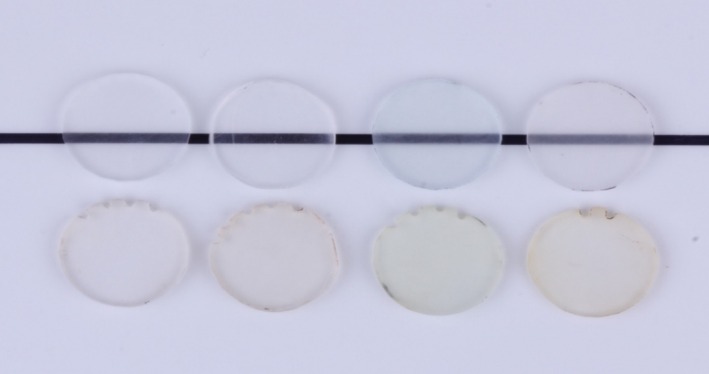
Photograph of representative specimens of (left to right): Milled, heat‐cured, KeySplint Soft, and NightGuard Flex before (top row) and after (bottom row) staining.

A photograph of representative specimens of the 3D‐printed materials with all different surface treatments and glycerin curing conditions is presented in Figure 5. A photograph of representative specimens of polished specimens from different materials is presented in Figure 6. Photographs of pre‐stained specimens were taken against a black line to demonstrate translucency and next to stained specimens to demonstrate stain resistance.

**FIGURE 7:**
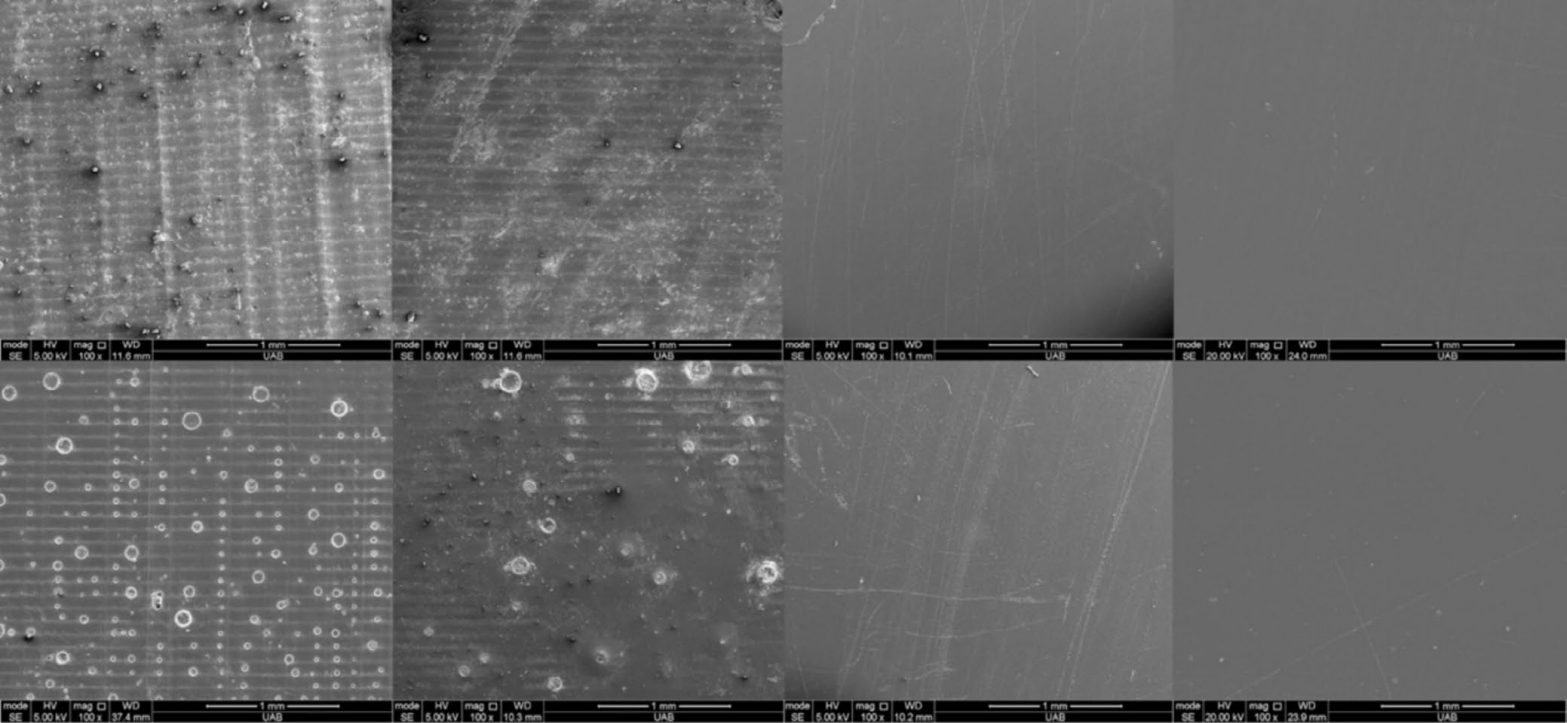
SEM images of representative specimens of KeySplint Soft (left to right): As‐printed, optical polish, resin‐coated, and polished after curing in air (top row) or glycerin (bottom row).

**FIGURE 8:**
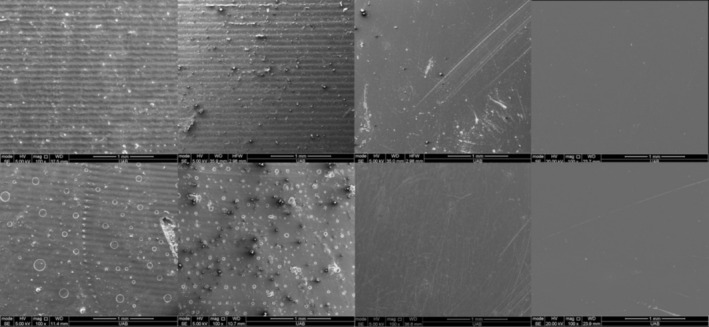
SEM images of representative specimens of NightGuard Flex (left to right): As‐printed, optical polish, resin‐coated, and polished after curing in air (top row) or glycerin (bottom row).

Representative SEM images of all KeySplint Soft materials are presented in Figure 7 and NightGuard Flex 2 materials are presented in Figure 8. Specimens from the as‐printed and optical polish group demonstrated printing lines, whereas the resin‐coated and polished groups did not. As‐printed and optical polish specimens cured in glycerin have small bubbles present on their surface.

## 4 Discussion

Improving the translucency and stain resistance of an occlusal device can improve its esthetic appearance. Decreasing roughness is not only important to help with these esthetic properties, but it can also improve comfort and decrease plaque accumulation [10, 11]. This study found significant differences in the roughness, translucency, and stain resistance of both 3D‐printed occlusal device materials for different surface treatments and glycerin curing conditions. Therefore, the first null hypothesis was rejected. For both materials, surface roughness was lowest and translucency was highest in the polished specimens, followed by resin‐coated, optical polished, and as‐printed specimens. Curing in glycerin improved staining for both materials for all surface treatments other than polishing.

The roughness of the as‐printed specimens (Ra = 2.91–3.41 μm for KeySplint Soft and 2.47–2.67 μm for NightGuard Flex) is similar to a previously reported Ra value for KeySplint Soft printed at 90° to the build plate (Ra = 2.16 μm) [7]. In the previous study, printing at 0° and 45° to the build plate resulted in lower Ra values of 0.34 and 1.28 μm, respectively [7]. The choice to print at 90° in the current study was to prevent supports occurring on the portion of the disc that would be used for translucency and staining measurement. The roughness of the specimens printed in the optical polish tank was lower than that of the standard tank. These results were expected because of the blurring between voxels that could occur because of the intensity‐controlling film on the resin tank. Observation of the SEM images of the specimens printed in the optical polish tank (Figures 7 and 8) shows a lower intensity of the print lines. The slightly lower roughness observed for KeySplint Soft (as‐printed and optical polish) specimens when curing in glycerin was not expected and cannot be explained. SEM images of these specimens reveal less intense print lines for these specimens (Figures 7 and 8).

The roughness of the resin‐coated specimens was significantly lower than the as‐printed and optical polished specimens; however, it was higher than the polished specimens. A previous study of 3D‐printed denture base materials also reported a greater surface roughness of resin‐coated specimens (Ra = 0.16 μm) than polished specimens (Ra = 0.03 μm) [15]. Despite the slight difference in roughness, the SEM images of both resin‐coated and polished surfaces appear smooth (Figures 7 and 8). Additionally, the roughness levels of the resin‐coated specimens (Ra = 0.27–0.28 μm for KeySplint Soft and 0.22 μm for NightGuard Flex) are below the threshold for which the tongue can perceive (0.5 μm) [9] and near the threshold at which biofilm may form (0.2 μm) [10].

For translucency testing, polishing allowed the highest translucency for both materials. Polished specimens created a more translucent surface than resin‐coated specimens (Figure 5) despite both materials appearing microscopically smooth (Figures 7 and 8). One possible explanation for this observation is that the presence of any voids at the interface between the as‐printed surface and the overlaying resin coating may affect translucency. Additionally, KeySplint Soft specimens with a resin coat had a slight yellow hue that likely decreased translucency. Without resin coating, KeySplint Soft typically develops a slight yellow hue after curing that diminishes after 2 days. The yellow hue could be due to a chromophore in a light initiator in the resin. The residual yellow hue present in the resin‐coated specimens could be due to insufficient photobleaching of the initiator chromophore as the coating would not have received any light polymerization within the 3D printer. 3D‐printed resins achieve about 50% of the degree of conversion within the printer [16]. A final explanation is that the resin‐coated specimens were slightly thicker than the polished specimens (up to 0.5 mm on each side) due to the addition of material with resin coating and removal of material with polishing.

The optical polish tank allowed a more translucent surface than printing in the standard tank (as‐printed group) for most conditions, which is due to the less intense printing lines present on the specimens (Figures 7 and 8). As noted previously, curing in glycerin created a smoother surface for the KeySplint Soft material (as‐printed group) which may explain the slightly greater translucency for this material when cured in glycerin.

For staining, the most dramatic difference was observed between specimens that were cured in air compared to those cured in glycerin. If oxygen is present during the final cure of a resin, it can react with free radicals necessary for chain reactions between polymers. As a result, the outer layer of the resin is incompletely polymerized and susceptible to staining by reacting with pigments [16]. Glycerin acts as an oxygen‐shielding agent when curing 3D‐printed resins and prevents the formation of the outer oxygen‐inhibition layer. The observation of small bubbles on the surface of the as‐printed and optical polish groups suggests that some air present in the 3D‐printed samples must have migrated to the surface of specimens during curing and become trapped under the layer of glycerin. The absence of the bubbles in the resin‐coated groups is likely due to the absence of air in the resin coating layer. The polished specimens did not require curing in glycerin to minimize staining as polishing would remove the oxygen‐inhibition layer.

Without glycerin curing, the resin‐coated group underwent more staining than the polished specimens despite both surface treatments creating a smooth surface (Figures 7 and 8). The increased staining of the resin‐coated group could be due to a lower degree of conversion of the resin as the resin lacked curing in the printer.

In the second part of the study, the 3D‐printed materials underwent more staining than the milled material, and one of the materials underwent more staining than the heat‐cured material. Therefore, the second null hypothesis was rejected. The 3D‐printed materials had a similar roughness to the milled material and lower roughness than the heat‐cured material; therefore, the increased staining would not be credited to the surface roughness. It is possible, then, that the increased staining could be credited to voids between 3D‐printed layers and a lower degree of resin conversion [6]. No voids could be visualized on the surface of the 3D‐printed materials using SEM.

There were several limitations in this study. First, the study considered only specimens printed at 90° to the build plate. Printing at 0° and 45° to the build plate would decrease the roughness of the as‐printed specimens. Additionally, other printing angles may have affected the benefits of printing with an optical polish tank. Printing at 90° was chosen to prevent the presence of supports on the flat surfaces of the specimens. Newer software updates, however, allow printing specimens at angles without the need for supports. Future studies could examine the effect of optical polish tanks at different print angles. Second, as previously mentioned, the resin‐coated specimens were thicker than the milled specimens, which could have decreased their translucency. The increased thickness from the application of a resin coat is unavoidable clinically. Third, the statistical outcomes may have been affected due to the power of the study. Future studies may consider other oxygen‐shielding agents such as curing in a vacuum or in nitrogen. Future studies may also evaluate the efficacy of agents for cleaning occlusal device materials that have undergone staining and their effect on the properties of the material. Finally, future studies may examine the ability of different surface treatments to maintain esthetic properties following tooth wear, thermocycling, or acidic beverage exposure.

## 5 Conclusion

Within the limitations of this current study, it was concluded that polishing 3D‐printed occlusal device materials generally produces the smoothest, most translucent, and stain‐resistant surface. Application of a resin coat, however, provides a similar level of roughness and improvements in translucency with a more efficient workflow. If a resin coat is used, a final cure in glycerin is applied to prevent excess staining. The internal surface of an occlusal device is not polished, and therefore, a final cure in glycerin is recommended to prevent staining of the device.

## Conflicts of Interest

Dr. Lawson has received research grants and speaking honoraria from Dentsply Sirona and Ivoclar Vivadent. The other authors declare no conflicts of interest.

## Data Availability

The data that support the findings of this study are available from the corresponding author upon reasonable request.
